# Gradient Boosting Prediction of Overlapping Genes From Weighted Co-expression and Differential Gene Expression Analysis of Wnt Pathway: An Artificial Intelligence-Based Bioinformatics Study

**DOI:** 10.7759/cureus.67207

**Published:** 2024-08-19

**Authors:** Pradeep Kumar Yadalam, Ramya R, Raghavendra Vamsi Anegundi

**Affiliations:** 1 Periodontics, Saveetha Dental College, Saveetha Institue of Medical and Technical Sciences (SIMATS) Deemed University, Chennai, IND; 2 Oral Pathology and Oral Biology, Saveetha Dental College, Saveetha Institue of Medical and Technical Sciences (SIMATS) Deemed University, Chennai, IND

**Keywords:** artificial intelligence, gene, bone formation, wnt pathway, weighted co-expression network analysis

## Abstract

Introduction

The Wnt (wingless-related integration site) signalling pathway is crucial for bone formation and remodelling, regulating the commitment of mesenchymal stem cells (MSCs) to the osteoblastic lineage. It triggers the transcriptional activation of Wnt target genes and promotes osteoblast proliferation and survival. Weighted co-expression network analysis (WGCNA) and differential gene expression analysis help researchers understand gene roles. Gradient boosting, a machine learning technique, enhances understanding of genetic and molecular mechanisms contributing to overlap genes, improving gene regulation and functional genomics. The aim is to predict overlapping genes in the Wnt signalling pathway.

Methods

Differential gene expression analysis was performed using the National Center for Biotechnology Information (NCBI) geo dataset-GSE251951, focusing on the effect of Wnt signaling on treatment. The WGCNA module was analyzed using the iDEP tool to identify interconnected gene clusters. Hub genes were identified by calculating module eigengenes, correlated with external traits, and ranked based on module membership values. The study utilized gradient boosting, an ensemble learning method, to predict models, evaluate their performance using metrics like accuracy, precision, recall, and F1 score, and adjust predictions based on gradient and learning rate.

Results

The dendrogram uses the "Dynamic TreeCut" algorithm to analyze gene clusters, aiding researchers in understanding gene modules and biological processes, identifying co-expressed genes, and discovering new pathways. The confusion matrix displays 88 actual and predicted cases. The gradient boosting model achieves 78.9% accuracy in predicting Wnt pathway overlapping genes, with a respectable area under the curve (AUC) and classification accuracy values. It accurately predicts 73.9% of samples, with a high precision ratio and low recall.

Conclusion

Future research should enhance differential expression analysis and WGCNA to identify key Wnt pathway genes, improve sensitivity, specificity, hyperparameter tuning, and validation experiments, and use larger datasets.

## Introduction

The Wnt (wingless-related integration site) signalling pathway is a crucial part of bone formation and remodelling, regulating the commitment of mesenchymal stem cells (MSCs) to the osteoblastic lineage, osteoblast proliferation, and differentiation. It binds Wnt ligands to cell surface receptors, stabilizing and nuclear translocating β-catenin, a cytoplasmic protein [1]. The Wnt pathway also promotes osteoblast proliferation and survival by stimulating the production of growth factors and cytokines and inhibiting apoptosis. It also influences osteoblast function by balancing osteoblast and osteoclast activity in bone remodelling, stimulating osteoprotegerin production, a decoy receptor that inhibits osteoclast differentiation and activity, thereby promoting bone formation. The Wnt pathway is crucial for bone formation, but its dysregulation can lead to pathological conditions. Mutations in *LRP5* or β-catenin can cause high bone mass disorders, while loss-of-function mutations can cause low bone mass disorders. The Wnt pathway promotes bone formation, osteoblast proliferation, survival, and function, highlighting its importance in bone biology [2].

Mesenchymal stem cells (MSCs) have the potential to differentiate into bone, cartilage, fat, tendon, and muscle tissues. They are harvested from the patient's body, especially from bone marrow, and have therapeutic potential in regenerative medicine. The Wnt signalling pathway plays a crucial role in promoting the osteogenic differentiation of MSCs [3]. The pathway inhibits adipogenic differentiation and upregulates osteogenic regulators, contributing to the progression of MSCs into mature osteoblasts. The noncanonical Wnt pathway also induces osteogenic differentiation through a different mechanism. Wnt pathways [4] and other signalling pathways regulate osteogenic differentiation in MSCs. BMPs can enhance or antagonize Wnt-induced differentiation, with BMP2, 6, and 9 major osteogenic growth factors [5]. Functional Wnt signalling is required for BMP-induced differentiation, and knocking out BMP receptor type 1 leads to increased bone mass. The inactivating mutation of *LRP5* causes osteoporosis pseudo glioma syndrome (OPPG), characterized by early-onset osteoporosis, low bone mineral density, and blindness. In mice, inactivating mutations impair fracture healing, while a gain-of-function missense mutation leads to high-bone-mass phenotypes. *LRP6* mutations severely affect osteogenic development in humans and mice, leading to osteoporosis, low BMD, neonatal death, and limb abnormalities [6].

Combining weighted gene co-expression network analysis (WGCNA) [7] and differential gene expression (DGE) analysis is a powerful method for understanding complex biological processes, identifying gene overlap, and understanding gene regulation networks. WGCNA and DGE analysis are powerful tools for analysing high-dimensional gene expression data. WGCNA groups genes based on co-expression patterns [8], while DGE analysis identifies differential expression between conditions or phenotypes potentially associated with specific biological functions. WGCNA and DGE analysis can be integrated by identifying gene overlaps between WGCNA-identified modules and differentially expressed genes. These are crucial regulators or functional drivers of biological processes, demonstrating significant changes in expression levels across conditions.

WGCNA and DGE analysis enable researchers to understand overlapping genes' functional roles, enabling functional enrichment analysis like gene ontology or pathway analysis, and providing insights into biological processes. Integrating WGCNA and DGE analysis can reveal regulatory relationships between overlapping genes. WGCNA creates co-expression networks, while DGE analysis incorporates differential expression information. This helps identify specific regulatory relationships for conditions or phenotypes, revealing key factors driving biological processes [9]. Overlap genes are shared genes in biological processes or molecular pathways. Predicting overlap genes can provide insights into functional relationships. Gradient boosting, a machine learning technique, combines multiple weak predictive models to create a strong predictive model. This approach helps researchers understand genetic and molecular mechanisms contributing to overlap genes, providing valuable insights for complex biological processes. It enhances understanding of functional relationships and regulatory networks among genes, improving gene regulation and functional genomics. So, we aim to predict the overlapping genes in the Wnt signalling pathway from WGCNA and differentially expressed genes using gradient boosting.

## Materials and methods

This computational study was conducted at Saveetha Dental College, Chennai, India between May 1 and May 31, 2024. This study employed a computational approach to investigate the potential of Wnt signaling in osteoporosis treatment.

DGE analysis

Using the National Center for Biotechnology Information (NCBI) geo dataset GSE251951 [10], DGE was performed using the Gene Expression Omnibus (GEO) tool. The dataset reveals whether Wnt signaling can induce effective osteoporosis treatment, promoting bone formation through aerobic glycolysis in the *Mus musculus* in a computational model. Datasets were divided into nonexposed to wnt3a and exposed to wnt3a and DGE. The results were analyzed for differentially expressed genes, fold changes, p-values, and adjusted p-values.

WGCNA

The WGCNA module used the iDEP tool [11], a standardized gene expression dataset, to identify highly interconnected gene clusters, ensuring comparable gene and sample distributions. WGCNA calculates pairwise gene correlations, constructing an adjacency matrix and transforming it into a topological overlap matrix (TOM), measuring gene interconnectedness within a network [12]. The TOM generates a hierarchical clustering tree (dendrogram) using the average linkage method, grouping genes with similar expression patterns indicating potential functional relationships. The Dynamic TreeCut algorithm is used to identify distinct modules or clusters within the co-expression network, with a minimum module size parameter of 30 genes. Identifying modules allows for the characterization and analysis of their biological relevance through gene ontology, biological pathways, or functional annotations of genes within each module. The WGCNA analysis in iDEP aids researchers in identifying gene relationships, functionally related groups, regulatory mechanisms, key genes, and modules associated with specific biological processes or diseases.

Identification of hub genes

In WGCNA, hub genes are highly connected genes within a module that are crucial for the module's functioning and may have key roles in the biological process or disease being studied. To identify hub genes, we have to calculate module eigengenes (MEs) for each module, correlate them with external traits, identify the module with the highest correlation, extract gene membership and module eigengene values, rank genes within the module based on module membership (MM) values, and further characterize hub genes by examining their functional annotations, gene ontology terms, or biological pathways.

Identification and prediction of overlap genes

Top hub genes from WGCNA and DGE were tabulated, and the prediction model was performed using gradient boosting. The model was trained sequentially on the training data to predict overlap genes using gradient boosting, learning from previous mistakes to improve predictive accuracy. The model's performance could be evaluated using accuracy, precision, recall, and F1 score metrics. The model's importance could be understood by interpreting its feature importance scores. Finally, the model could predict new data, identifying overlap genes based on the selected features and learned importance. The data was divided into training (80%) and test (20%) datasets, respectively. Preprocessing steps such as outliers' removal and data normalization were applied.

Gradient boosting architecture

Gradient boosting is an ensemble learning method that combines multiple weak learners, often in the form of decision trees. A loss function is used to quantify the difference between predicted and actual values. Gradient descent optimization minimizes the loss function, and the model adjusts predictions based on the gradient and learning rate. The learning rate controls the contribution of each base model to the ensemble, and regularization techniques like shrinkage or dropout can be applied to avoid overfitting and improve generalization. Feature importance scores indicate the relative importance of each feature in making predictions.

## Results

**Figure 1:**
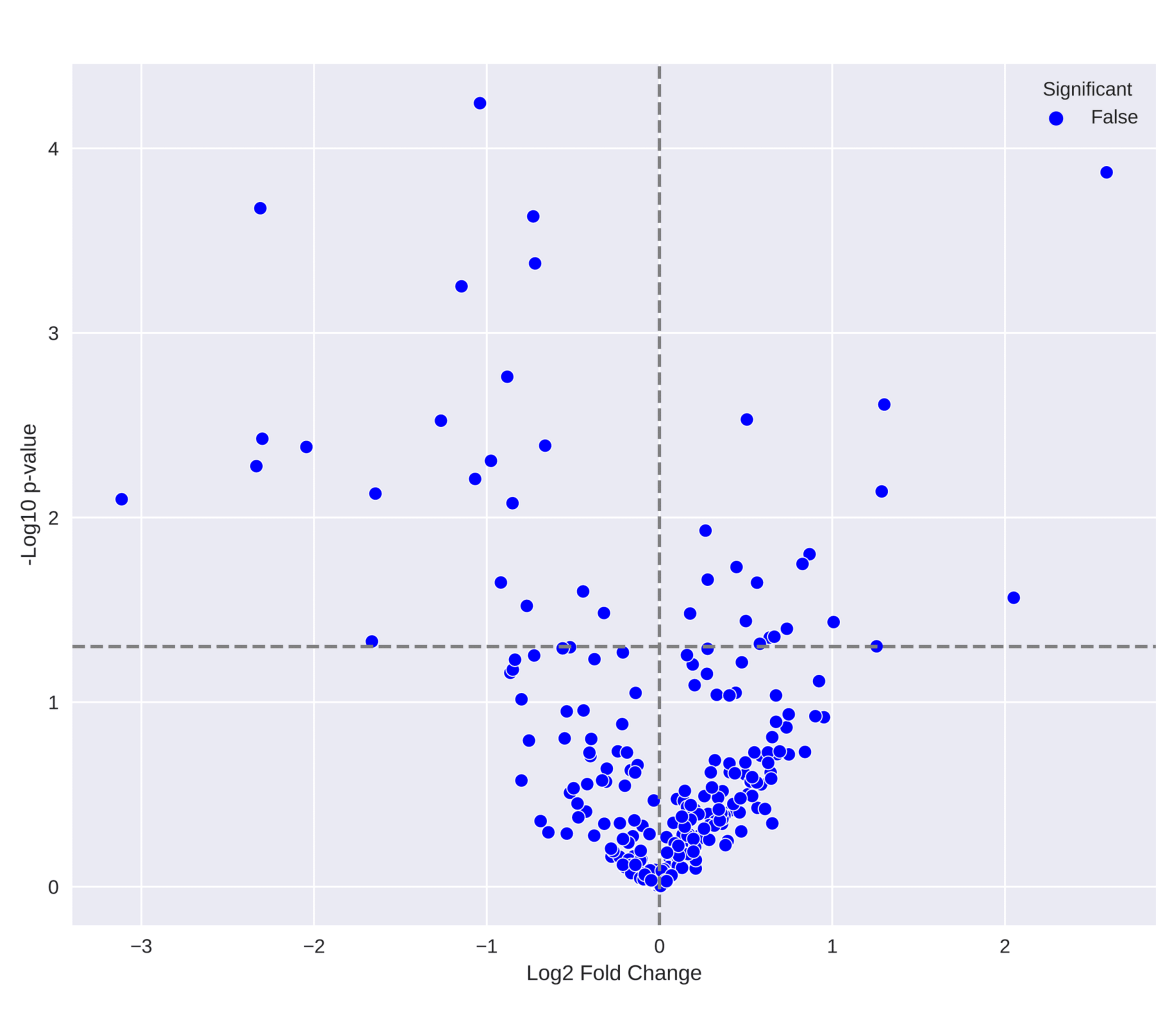
Volcano plot of the top 250 differential gene expressions X-axis represents log2 fold change and the y-axis represents -log10 p-value. Statistically significant genes have a -log10 p-value greater than 1.3, while upregulated genes have a log2 fold change greater than 1.5.

**Figure 2:**
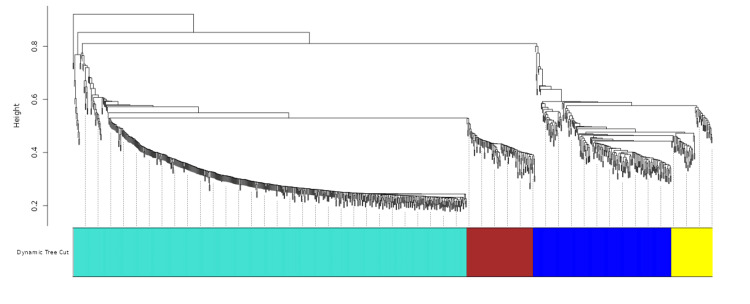
Gene dendrogram and module colors This image shows how genes are grouped based on their similarity. Genes with similar characteristics are clustered together. The height of the branches indicates how different the gene groups are. The colored blocks at the bottom represent different groups of genes.

**Figure 3:**
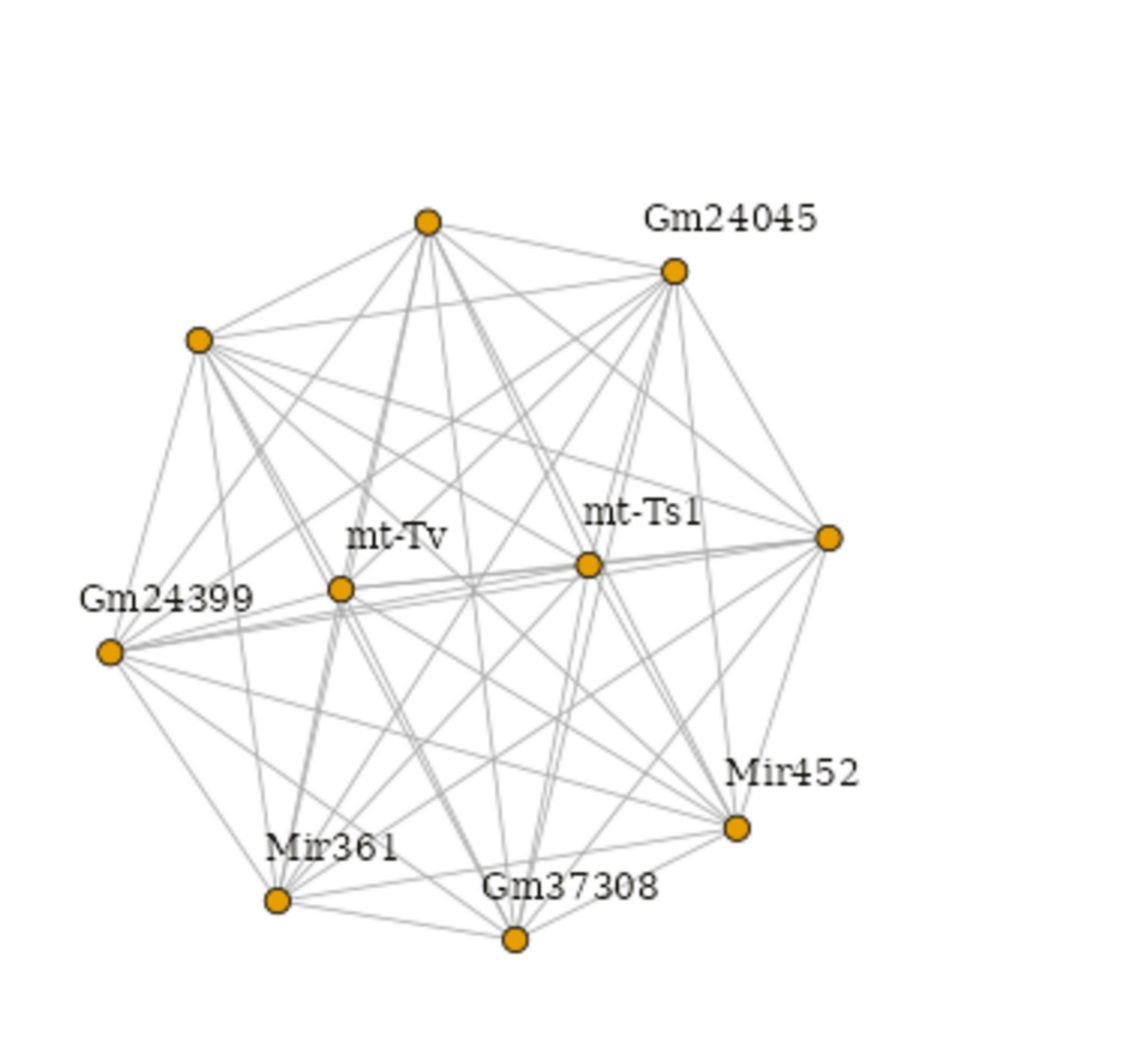
Graphical representation of relationships between various entities, typically used to visualize interactions within a system.

The 249-row dataset exhibited a broad spectrum of up- and down-regulated genes. While statistically significant differentially expressed genes were identified at a conventional threshold, the relatively low number suggests further exploration may be warranted, as depicted in Figure 1. WGCNA provided a hierarchical overview of gene expression patterns, a topological overlap matrix for quantifying gene interrelationships, and a soft thresholding approach to convert raw co-expression values into a weighted adjacency matrix. These outputs facilitate the identification of gene modules and associated biological processes. Dendrograms enabled the analysis of gene clusters, delineated by a Dynamic TreeCut algorithm. The colour-coded bar is segmented into four colours (Figures 2, 3) that represent distinct gene groups based on expression patterns, aiding in the comprehension of functional relationships, co-expressed gene identification, and the potential discovery of novel pathways or regulatory mechanisms.

This analysis helps understand functional relationships, identify co-expressed genes, and potentially discover new pathways or regulatory mechanisms in biological systems. Network nodes labelled with identifiers like Gm24045, mt-Tv, mt-Ts1, Gm24399, Mir361, Mir152, and Gm37308 suggest they could represent biological genes or molecular entities. Edges, lines connecting nodes, indicate relationships or interactions. Density indicates strong or multiple interactions, while sparser connections indicate less interaction. Network analysis requires selecting an optimal soft threshold power, typically above 0.8 or 0.9, to construct meaningful biological networks from gene expression data, identifying co-expressed gene modules and key drivers.

**Figure 4:**
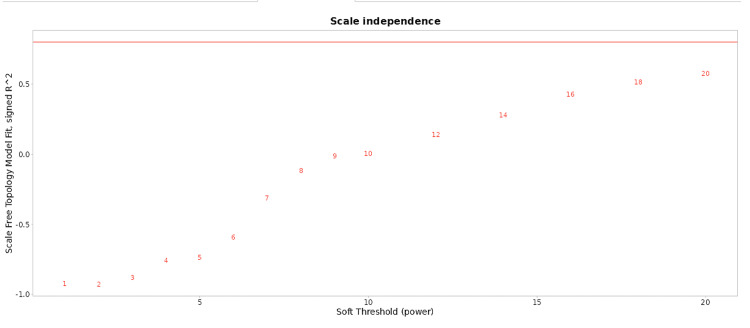
Scale independence Scale Independence plots the scale-free topology model fit (y-axis) against the soft threshold power (x-axis). The x-axis represents the soft threshold power, a parameter used in network analysis, particularly in WGCNA, to highlight stronger correlations between nodes. The y-axis indicates the scale-free topology model fit, with a higher R^2^ value indicating better conformity to a scale-free topology, a crucial assumption in network analysis methods. The graph shows that as soft threshold power increases, the scale-free topology model fit initially increases, peaking and then stabilizing or slightly declining. WGCNA: weighted gene co-expression network analysis

**Figure 5:**
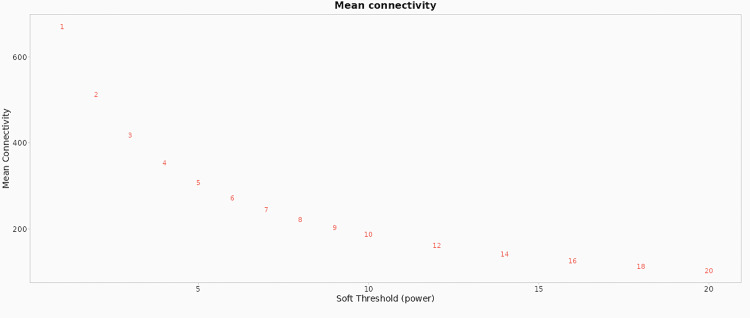
Scale-free topology model fit The x-axis, a parameter in network analysis, ranges from 1 to 20, highlighting stronger correlations between nodes and minimizing weaker ones. The graph shows the scale-free topology model fit, with a higher R^2^ value indicating better conformity. Data points are labeled with soft threshold power numbers, and the graph helps select an optimal threshold power for the best scale-free properties.

Network nodes, labelled with identifiers such as Gm24045, mt-Tv, mt-Ts1, Gm24399, Mir361, Mir152, and Gm37308, represent biological genes or molecular entities. Edges connecting these nodes signify relationships or interactions. Node density indicates the strength or frequency of interactions. To construct meaningful biological networks from gene expression data, an optimal soft threshold power, typically exceeding 0.8 or 0.9, is essential for identifying co-expressed gene modules and key regulatory elements, as visualized in Figures 4, 5.

The gradient boosting model performed well in predicting the target variable, with an area under the curve (AUC) value of 0.789 and a classification accuracy of 0.739. However, the model's accuracy is limited by the specific domain and context of the problem. The model's F1 score of 0.706 indicates a balanced trade-off between precision and recall, with a precision value of 0.749 indicating a low false positive rate, resulting in 75% accuracy. The model's recall value of 0.739 indicates a reasonable ability to identify positive instances, identifying approximately 74% of the actual positive instances in the dataset. The gradient boosting model, with a specificity value of 0.564, shows moderate accuracy, precision, recall, and F1 score, but struggles with identifying negative instances. Improvement in specificity is needed, potentially through adjusting thresholds or exploring other models. (Table 1) 

**Table 1:** Classification model performance AUC: area under the curve; CA: classification accuracy; F1: F-score

Model	AUC	CA	F1	Precision	Recall	Specificity
Gradient Boosting	0.789	0.739	0.706	0.749	0.739	0.564

**Figure 6:**
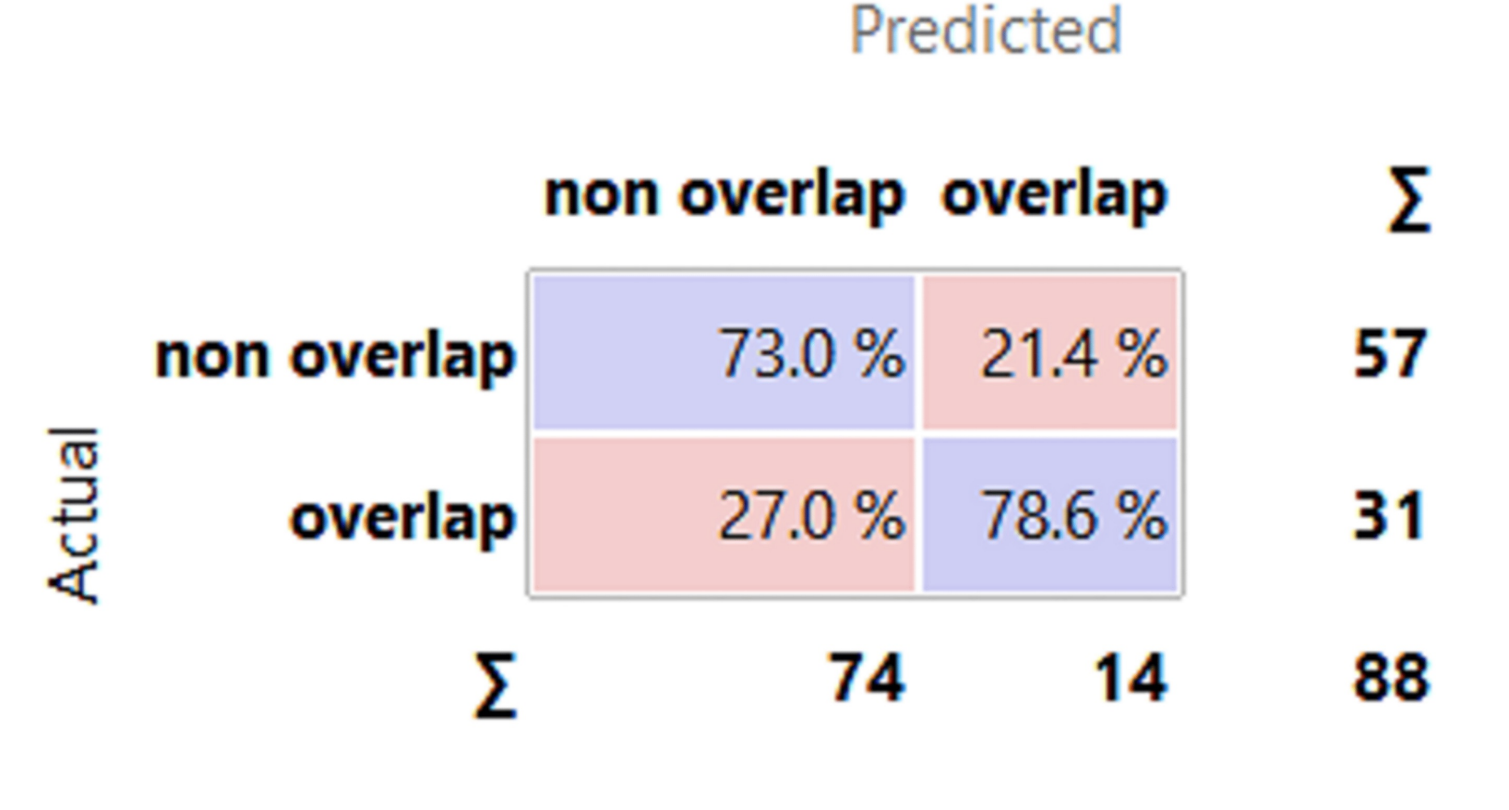
Confusion matrix

The confusion matrix displays 88 actual and predicted cases, assessing the model's performance in distinguishing between "non-overlap" and "overlap" categories and identifying strengths and weaknesses in sensitivity and specificity (Figure 6). The true negative cell shows that the model correctly predicted "non-overlap" 73.0% of the time, about 42 cases out of 57. The model's false positive and false negative cells indicate that it incorrectly predicted "overlap" and "non-overlap" cases, respectively, at 21.4% and 27.0% of the cases.

## Discussion

Recent research on the canonical Wnt pathway has provided new insights into regulating this pathway. Activation of the canonical Wnt pathway leads to the accumulation and movement of β-catenin into the nucleus, activating transcription factors that control specific genes involved in cellular development. The intracellular signaling of Wnt is complex due to the involvement of multiple Fz receptors and the recently established role of LRP5 and LRP6 as co-receptors for Wnt proteins [13]. The Wnt pathway is a promising therapeutic target for bone repair and skeletal homeostasis, with abnormalities in Wnt/β-catenin signaling implicated in osteoarthritis. Sclerostin, a product of the *SOST* gene, inhibits Wnt signaling and is being investigated for osteoporosis [14]. Dual inhibition of Wnt and sclerostin antibody treatment results in synergistic bone formation. Dual inhibition of Wnt and sclerostin antibody treatment results in synergistic bone formation. This treatment is being explored in clinical trials for various medical conditions. In this study, we analyzed weighted co-expression analysis and differential gene expression of the Wnt-based pathway.

Limited research exists on identified hub nodes (Gm24045, mt-Tv, mt-Ts1, Gm24399, Mir361, Mir152, and Gm37308) in the Wnt pathway and bone formation of mice. These non-coding genes may have regulatory functions, but their specific roles remain unclear. mt-Tv and mt-Ts1 are transfer RNAs involved in mitochondrial function and energy production, while Mir361 and Mir152 are microRNAs [15] that regulate gene expression [4]. Further studies are needed to understand their functions and potential contributions, similar to studies that identified predictive hub genes. Six feature genes (*AADAT, APOF, GPC3, LPA, MASP1*, and *NAT2*) were identified using machine learning algorithms such as Random Forest, support vector machine-recursive feature elimination [9,16], and one more study similar to the four-gene model was developed for diagnosing sepsis/severe acute respiratory distress syndrome (ARDS), demonstrating high diagnostic and predictive performance through calibration curves and decision curve analyses [8,9,17].

Future research should investigate differentially expressed genes to identify key genes and pathways in the Wnt pathway. WGCNA can provide insights into gene expression patterns and functional relationships as shown in Figures 2-5. Network analysis can help understand interactions between genes and regulatory mechanisms. Future directions should focus on improving the model's sensitivity and specificity, tuning hyperparameters, and conducting comprehensive validation experiments. However, limitations include the dependence on dataset quality and the potential for generalization to other biological systems.

The gradient boosting model achieves 78.9% accuracy in predicting Wnt pathway overlapping genes, with respectable AUC and CA values. It accurately predicts 73.9% of samples, with a high precision ratio and low recall, as shown in Figure 6. Its high precision ratio indicates low false positives and a low recall ratio, suggesting accurate predictions. However, the model's lower F1 score suggests it needs improvement in balancing precision and recall. Future improvements could involve improving the recall rate, reducing false negatives, and capturing more true overlapping genes. The quality of training data and gene annotations limits the model's performance. Obtaining a larger dataset and conducting further experimental validations could enhance its performance.

## Conclusions

The present study aimed to elucidate the intricate regulatory mechanisms within the Wnt signaling pathway, a key determinant of osteogenesis. By integrating DGE analysis and WGCNA, the current study identifies pivotal gene modules and their potential roles in bone formation. The application of gradient boosting provided a computational framework for predicting gene interactions within this pathway.

While our findings offer preliminary insights into the complex architecture of the Wnt signalling network, further research is imperative to validate these observations in larger and more diverse cohorts. A deeper understanding of the identified gene modules and their functional implications is essential for translating these findings into clinically relevant applications. Ultimately, unravelling the complexities of Wnt signalling holds the potential to inform the development of novel therapeutic strategies for bone-related disorders.
